# Melatonin and non-small cell lung cancer: new insights into signaling pathways

**DOI:** 10.1186/s12935-019-0853-7

**Published:** 2019-05-16

**Authors:** Mohammad Hossein Pourhanifeh, Mehran Sharifi, Russel J. Reiter, Abdoulhossein Davoodabadi, Zatollah Asemi

**Affiliations:** 1 Research Center for Biochemistry and Nutrition in Metabolic Diseases, Kashan University of Medical Sciences, Kashan, Iran; 2 Department of Hematology and Oncology, School of Medicine, Isfahan University of Medical Sciences, Isfahan, Iran; 3 Department of Cellular and Structural Biology, University of Texas Health Science Center, San Antonio, TX USA; 4 Departments of General Surgery Trauma Research Center, Kashan University of Medical Sciences, Kashan, Iran

**Keywords:** Melatonin, Lung cancer, Malignancy, Inflammation, Gene expression

## Abstract

Non-small-cell lung cancer (NSCLC) is a type of malignancy with progressive metastasis having poor prognosis and lowered survival resulting from late diagnosis. The therapeutic approaches for the treatment of this incurable cancer are chemo- and radiotherapy. Since current treatments are insufficient and because of drug-induced undesirable side effects and toxicities, alternate treatments are necessary and critical. The role of melatonin, produced in and released from the pineal gland, has been documented as a potential therapy for NSCLC. Melatonin prevents tumor metastasis via inducing apoptosis processes and restraining the autonomous cell proliferation. Moreover, melatonin inhibits the progression of tumors due to its oncostatic, pro-oxidant and anti-inflammatory effects. As a result, the combined treatment with melatonin and chemotherapy may have a synergistic effect, as with some other tumors, leading to a prolonged survival and improved quality of life in patients with NSCLC. This review summarizes the available data, based on the molecular mechanisms and related signaling pathways, to show how melatonin and its supplementation function in NSCLC.

## Introduction

Lung cancer is a leading cause of cancer-related mortality and morbidity in the global population [1]. Histologically, there are two types: small-cell lung cancer and non-small-cell lung cancer (NSCLC). Approximately, 85% of lung cancer cases are classified as NSCLC, and the majority of these patients present at advanced stage at the time of diagnosis [1, 2]. NSCLC comprises three subtypes including adenocarcinoma, squamous cell carcinoma and large-cell carcinoma. NSCLC has poor prognosis and based on the International Staging System for Lung Cancer, is related to locally advanced or metastatic disease with stage IIIB and stage IV [3]. The expected median survival of NSCLC is 6 months and a 5-year survival of 2% is anticipated [4]. Moreover, the symptoms of the disease such as chest pain, hoarseness, weight loss, coughing and wheezing can significantly lower the functional independence of NSCLC patients [5, 6]. Chemotherapy a proven therapy for NSCLC and improves patient’s survival. However, it often has treatment-associated toxicity that reduces its therapeutic potential. Thus, searching for alternative and complementary treatments, to reduce the adverse effects and enhance the therapeutic properties of chemotherapy, is an essential issue.

As a neurohormonal agent, melatonin (*N*-acetyl-5-methoxytryptamine) is a highly investigated anticancer agent which is endogenously synthesized and secreted by the pineal gland. Based on its circadian rhythm, the secretion of melatonin primarily occurs during night hours [7]. Melatonin is also produced by the other organs such as skin [8], gastrointestinal tract [9], lymphocytes [10], retina [11] and so forth [12]. Recently, some of the main roles of melatonin, including antioxidant, anxiolytic, antihypertensive and sedative effects have been demonstrated [13]. Also, a dose-dependent analgesic property of melatonin has been reported in experimental studies [14, 15]. The free radical scavenging capability of melatonin makes it an antioxidant agent in normal cells melatonin also has anti-inflammatory properties [16], while in cancer cells it has pro-oxidant activity [17, 18] and is involved in immune responses [19]. Numerous studies have reported positive effects of melatonin in the therapy of cancer. Previous non-clinical trials have shown that melatonin inhibits cell proliferation of tumor cells [7, 20, 21] and many clinical trials have documented that melatonin has beneficial effects on the survival in patients with cancer [22–24]. Additionally, melatonin has clinical benefits in gastric cancer [25], non-small-cell lung cancer [26], head and neck cancer [27] and the other cancers [28]. To evaluate the role of melatonin in NSCLC, based on the published data, the present review assesses the current knowledge of melatonin effectiveness as a potential treatment for NSCLC. In this review, we discuss multiple anticancer properties of melatonin, including anti-metastatic, tumor growth inhibition, apoptosis induction, anti-inflammatory, anti-proliferative, antioxidant, and its role in reducing the chemo-radiotherapy side-effects and increasing patient’s survival.

### Melatonin, a metastasis inhibitor in NSCLC

**Fig. 1:**
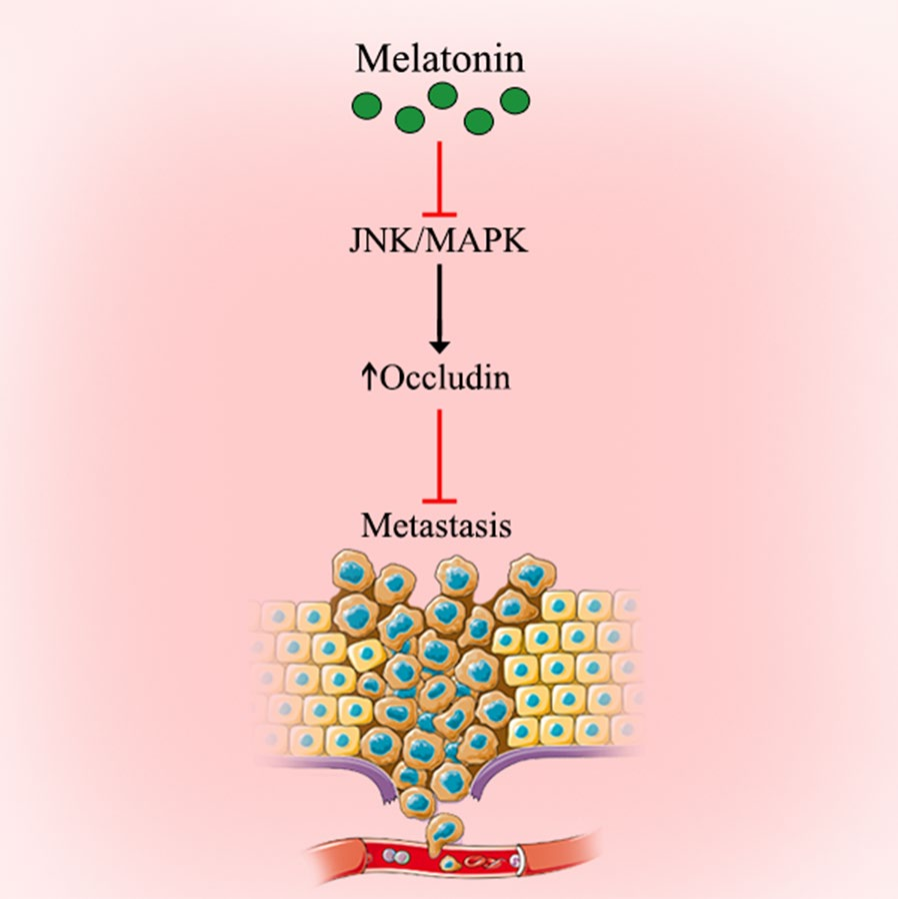
Schematic representation in targeting different signaling pathways using melatonin and its metabolites as a novel therapeutic strategy in the treatment of lung cancer

The migration of tumor cells, known as metastasis, is a process that worsens the stage of cancer due to its invasion of the surrounding tissues and other organs. Metastasis lowers the disease prognosis as well as patient’s the survival time [29]. Melatonin is capable of restraining the proliferation of tumor cells and inhibits their autonomous growth. Additionally, melatonin selectively blocks the signal transduction of pathways of tumor cells and especially those involved in metastasis [30–32]. By regulating the structure formation of microtubule and microfilament, melatonin inhibits metastasis and invasive properties of tumors [33]. Also, melatonin typically prolongs the cell cycle and delays tumor cell mitosis and inhibits cancer cells from entering the S phase [34, 35]. Specifically, melatonin has been shown to inhibit proliferation of NSCLC cells, which are responsible for metastasis in advanced lung cancer [36]. In summary, melatonin prevents the progression of tumor cells and metastatic processes by interacting with intracellular mechanisms related to proliferation and the invasion (Fig. 1).

### Melatonin exerts its anti-metastatic roles through inhibiting the c-Jun N-terminal kinase (JNK)/mitogen activated protein kinase (MAPK) signaling pathway

Down-regulation of occludin, as a trans-membrane protein related to tight junctions (TJ), is associated with invasiveness, staging and the high potential of metastasis in epithelial cancers [37, 38]. TJ paracellular transportis modulated via MAPK signaling pathway through down- and up-regulation of the expression of the several TJ proteins [39]. JNK is activated in numerous tumor cells and may have some transforming activities in several oncogenes [40]. The dysfunction and expression of occludin proteins are correlated with metastasis and tumor development [41, 42]. Zhou et al. [36] showed that treatment with melatonin markedly increased the expression of occludin in A549 cells. This overexpression on the cell surface enhanced the tight connection between cells to restrain A549 cell metastasis. In addition, melatonin inhibited the JNK/MAPK pathway based on its anti-migration effect. Clearly, the inhibitory properties of melatonin in lung adenocarcinoma should be more carefully evaluated.

### Melatonin inhibits tumor growth by regulation of epidermal growth factor receptor (EGFR) in NSCLC

Epidermal growth factor receptor is a tyrosine kinase receptor overexpressed on the surface of NSCLC tumor cells [43]. It plays major functions in tumor cell survival and activated phosphorylation of EGFR leads to the phosphorylation of downstream proteins that contribute to metastasis, invasion, cell proliferation and inhibition of apoptosis [44]. The intracellular mutation of EGFR or its overexpression in NSCLC patients has been observed in 43–89% of cases [45]. Consequently, the signaling pathway related to EGFR is inhibited by blocking the receptor using anti-EGFR antibodies or small molecules restraining the EGFR tyrosine kinase [46]. Notably, the inhibitors of EGFR tyrosine kinase, for instance erlotinib and gefitinib, are a standard therapy to treat patients with advanced NSCLC [47, 48]. The inactivating effect of melatonin on the growth of circadian-dependent tumor cells is mediated by EGFR suppression [49, 50]. As mentioned earlier, melatonin participates in EGFR signaling pathway regulation and this inhibitory activity could be in focus to discover more effective drugs.

### Melatonin induces its apoptotic properties through regulating B cell lymphoma 2 (Bcl-2)/Bax balance

Bcl-2, known as a proto-oncogene, was initially discovered in a follicular B-cl and currently has been confirmed in numerous tumors. The Bcl-2 protein is situated in the mitochondrial inner membrane and suppresses intrinsic apoptosis through arresting the cell in the G0/G1 phase of the cell cycle to prolong the survival of the tumor cell [51, 52]. The biological roles of Bcl-2 protein correlate with the protection of cancer cells from apoptosis and medication-induced death [53]. This protein has been assessed in various cancers to clear the predictive and prognostic significances, including NSCLC [54].

**Fig. 2:**
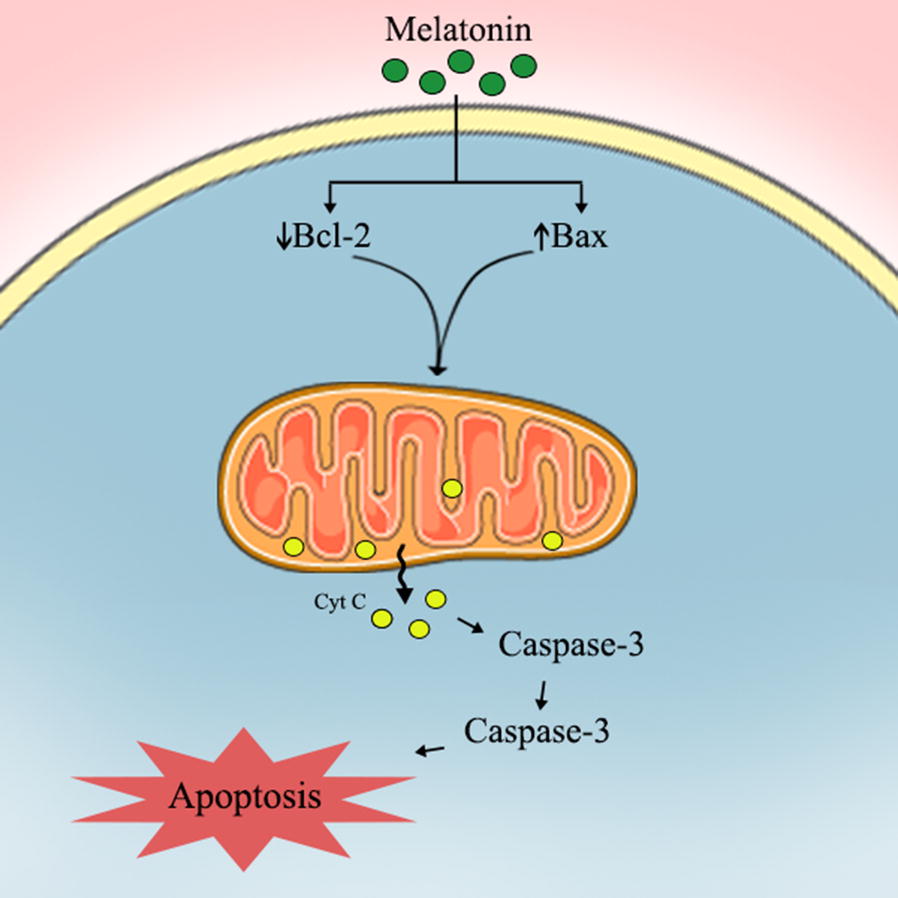
Proposed oncostatic actions of melatonin its metabolites on suppressing NSCLC cells metastasis

Similarly, Bax is a member of Bcl-2 family also located in mitochondria; it changes the permeability of its membrane to trigger caspases activation, leading to apoptosis [55, 56]. Bax proteins are expressed in all tumor cells, but their function is inactivated by Bcl-2 proteins [57, 58]. Melatonin induces the apoptosis in various tumors by regulating the apoptotic signaling pathway of Bcl-2 [59, 60]. For example, melatonin down-regulates the expression of Bcl-2 and up-regulates Bax expression leading to apoptosis in lung adenocarcinoma cells [61]. Pretreatment with melatonin enhances the berberine-mediated up-regulation of Bax proteins and down-regulation of Bcl-2 in lung cancer cells [62]. Also, melatonin decreases the phosphorylation of Bcl-2 in H1975 NSCLC resulting in apoptosis [63]. To whether melatonin and chemotherapy co-treatment is more effective in increasing the NSCLC patient survival via this apoptotic induction, clinical trials should be performed. In the identified in vitro study, pretreatment with melatonin effectively increased the berberine-induced downregulation of Bcl-2 and upregulation of the cleaved caspase-9, caspase-3, Bax and PARP as compared with treatment by berberine alone. This pretreatment also enhanced the cytochrome C release from a human lung cell line H1299 [62] (Fig. 2).

### Melatonin and its anti-inflammatory actions in relation to lung cancer

The cyclooxygenase 2 (COX-2) signaling is implicated in migration, growth and angiogenesis of lung cancer cells [64]. Its overexpression appears in different human cancers including cancer of the lung. The expression of COX-2 and the sequential production of prostaglandin E2 are capable of up-regulating phosphoinositide-3-kinase (PI3K), EGFR and ERK1/2 signaling to trigger cell proliferation, angiogenesis, metastasis and invasion of tumor cells [65]. Melatonin has anti-inflammatory and antitumor properties through inhibiting the expression of COX-2 [66]. The expression of COX-2 is controlled by the binding of several co-activators and transactivators to the corresponding sites on its promoter. Among all known regulatory agents distributing in the region of COX-2 transcription start site core promoter, the binding site of NF-κB is essential for the promoter activity of COX-2 [67, 68]. Melatonin may enhance the inhibition of COX-2 expression with this effect being mediated by stimulation of p50 nuclear factor (*NF*)-*κB* translocation from cell nuclei to cytosol [62].

The Raf/MEK/ERK and PI3K/Akt signaling also play critical roles in the growth of tumor cells and are implicated in the cancer-associated gene expression of COX-2 and human telomerase reverses transcriptase (hTERT). Pretreatment with melatonin (1.0 mM), in an in vitro investigation, significantly promoted the berberine-induced inhibition of Akt and ERK1/2 protein phosphorylation [62]. Therefore, Akt/ERK signaling should be considered as an important target for melatonin in increasing the inhibition of berberine-mediated growth in NSCLC cells and further studies are required. Caspase cascade activation is identified as an important basis of the apoptosis pathway. The release of cytochrome C from mitochondria into the cytosol is known as the precondition for caspase-dependent apoptosis.

### Melatonin inhibits the proliferation of tumor cells through suppressing activating enhancer-binding protein-2β (AP-2β)/hTERT signaling pathway

HTERT known as lung tumorigenesis hallmark, is highly expressed in the cancer cells of the lung; it is strictly regulated by AP-2β [69]. HTERT is a critical component of human telomerase which lengthens the ends of linear chromosome as well as maintaining their stability; this results in cellular immortalization [70]. HERT is overexpressed in variety cancers including lung cancer [71]. AP-2β displays its biological effects via the tumor-related gene hTERT activation. AP-2β is involved in different cell processes such as cell growth, apoptosis and the differentiation of tissue during embryogenesis. Melatonin reportedly downregulates AP-2β and hTERT expression, thereby suppressing cell proliferation [62]. Thus, the oncostatic action of melatonin includes inhibition of the progression of lung tumors via suppression the AP-2β/hTERT signaling pathway.

### Immunomodulatory role of melatonin in NSCLC

Melatonin also stimulates monocyte/macrophage, lymphocyte and natural killer cells to increase immunosurveillance. Moreover, lymphoid cells synthesize melatonin, this additional source of melatonin helps to regulate the human immune system by acting in an autocrine and paracrine manner [72]. Immunoenhancing ability of melatonin, with the enhancement of the production of pro-inflammatory cytokines including interleukin 1 (IL-1), IL-6, IL-12 and tumor necrosis factor-α (TNF-α) has been shown [73]. Furthermore, T-helper cells have a key role in protecting against malignancy, and melatonin also enhances the response of this cell by releasing IL-2, IL-10 and interferon gamma (IFN-γ) [74, 75]. Moreover, melatonin controls inflammation by inhibiting NF-kB. This leads to a reduced overproduction of leukocytes and pro-inflammatory cytokines [76]. Melatonin limits radiation-induced inflammation in the lung by decreasing oxidative stress and cytokines production [77].

### Melatonin, an oxidative stress regulator

In both in vivo and in vitro studies, melatonin protected healthy cells from treatment-related toxicity via its well documented antioxidant actions [78]. Toxicity, as a direct consequence of high levels of reactive oxygen species (ROS) [79, 80], causes oxidative damage to tumor and healthy cells, leading to unwanted side-effects. Melatonin is a powerful endogenous antioxidant in normal cells due to its ability to scavenge ROS, decrease the formation of free radicals, and activate antioxidant enzymes including glutathione peroxidase and superoxide dismutase [81, 82]. In cancer cells, melatonin might exert pro-oxidant effects [18]. Therefore, melatonin may inhibit cancer development [83]. The effect of melatonin in increasing the induction of oxidative stress in tumor cells, leading to induce cell death in lung adenocarcinoma, was shown by Fan et el. [61]. Enhancing the induction of oxidative stress in cancer may be an important oncostatic action of melatonin in NSCLC. Melatonin protects healthy cells against radiochemotherapy by reducing the overproduction of ROS [84]. For NSCLC cells, melatonin does not protect them from UV-induced apoptosis [85]. These combined abilities of melatonin should be used as a justification of this multifunctional agent a supplemental treatment for NSCLC.

### Melatonin declines the side-effects of chemotherapy and radiotherapy and increases survival

It has been shown that serum levels of melatonin and its metabolites, in comparison with those in healthy subjects, are lower in NSCLC patients [86, 87] and the concentrations further decreased due to standard chemotherapy [87]. Several studies have reported that melatonin reduces the chemotherapy-related side effects in patients and animals [23, 88]. Melatonin protects blood cells from radiation-induced and chemotherapy-related damages [74, 89]. Also, it lowers the frequency of chemotherapy-associated stomatitis, cardiotoxicity, asthenia and neurotoxicity [22]. Based on data from a meta-analysis of 21 randomised controlled trials (RCTs) conducted in patients with solid tumors, melatonin improves the frequency of partial and complete responses while reducing thrombocytopenia, leucopenia, nausea, hypotension and vomiting [90]. In addition to reduction of adverse events, adding melatonin to routine medical treatment may improve the survival in patients with NSCLC. Lissoni et al. [91] showed that melatonin in combination with cisplatin and etoposide significantly increased the survival time of NSCLC patients in a poor clinical state compared with chemotherapy alone. Also, chemotherapy was better tolerated in melatonin treatment individuals.

## Conclusions

**Fig. 3:**
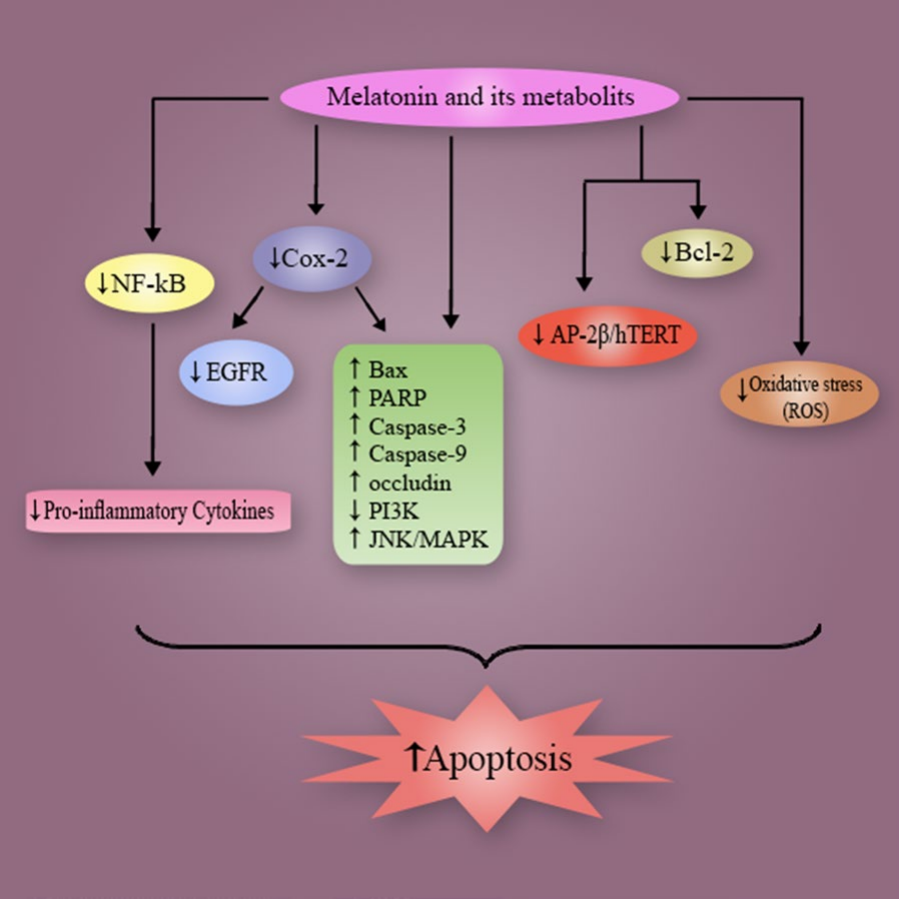
Proposed oncostatic actions of melatonin its metabolites on inducing cells apoptosis

Based on the published reports, melatonin is an appropriate complementary treatment that should be considered to enhance the clinical benefits in NSCLC therapy and to improve poor outcomes. Melatonin acts by affecting the related gene expression, apoptotic pathways and the proliferation of tumor cells (Fig. 3). In summary, several clinical trials have been designed to assess the potential beneficial effects of melatonin supplementation in the treatment of lung cancer and NSCLC, in particular. Additional RCTs should be carried out to determine the effectiveness of melatonin as a co-treatment for NSCLC.

## Data Availability

The primary data for this study is available from the authors on direct request.

## Abbreviations

NSCLC: non-small-cell lung cancer
EGFR: epidermal growth factor receptor
